# Gender differences in emotion perception and self-reported emotional intelligence: A test of the emotion sensitivity hypothesis

**DOI:** 10.1371/journal.pone.0190712

**Published:** 2018-01-25

**Authors:** Agneta H. Fischer, Mariska E. Kret, Joost Broekens

**Affiliations:** 1 University of Amsterdam, Department of Psychology, Amsterdam, the Netherlands; 2 Leiden University, Department of Psychology, Leiden, the Netherlands; 3 Delft University of Technology, Department of Intelligent Systems, Delft, the Netherlands; Radboud Universiteit, NETHERLANDS

## Abstract

Previous meta-analyses and reviews on gender differences in emotion recognition have shown a small to moderate female advantage. However, inconsistent evidence from recent studies has raised questions regarding the implications of different methodologies, stimuli, and samples. In the present research based on a community sample of more than 5000 participants, we tested the emotional sensitivity hypothesis, stating that women are more sensitive to perceive subtle, i.e. low intense or ambiguous, emotion cues. In addition, we included a self-report emotional intelligence test in order to examine any discrepancy between self-perceptions and actual performance for both men and women. We used a wide range of stimuli and models, displaying six different emotions at two different intensity levels. In order to better tap sensitivity for subtle emotion cues, we did not use a forced choice format, but rather intensity measures of different emotions. We found no support for the emotional sensitivity account, as both genders rated the target emotions as similarly intense at both levels of stimulus intensity. Men, however, more strongly perceived non-target emotions to be present than women. In addition, we also found that the lower scores of men in self-reported EI was not related to their actual perception of target emotions, but it was to the perception of non-target emotions.

## Introduction

The extent to which people are able to correctly perceive emotions on others’ faces has been regarded as one important ingredient of emotional intelligence [1]. Inferring information about the other’s thoughts, feelings and intentions is crucial in successful social interactions, and has for example been related to leadership skills [2,3] and satisfaction with social relationships [4]. Common sense tells us that women have better social skills, and are especially better at understanding of others’ emotions [5, 6, 7, 8, 9]. Indeed, research has shown that women often score higher on emotional intelligence or empathy tests than men, especially, but not only [10], if measured through self-reports, such as the Emotional Quotient Inventory (EQ-i [11]) the Empathy Quotient [12], the Interpersonal Reactivity Index (IRI) [13], or emotional awareness (LEAS)[14, 15].

One would expect that these beliefs and scores on self-report tests also reflect actual differences in emotion recognition performance, but there is debate on the question whether women outperform men on actual performance tests, for example in the recognition of emotions from the face. Although previous reviews and meta-analyses [16, 17, 18] have shown a small to moderate female advantage, recent studies have not always replicated this difference, leading to discussions about the extent to which and the circumstances in which women would outperform men, and how this should be explained [19]. Several explanations have been advanced, such as the idea that women would be particularly better in recognizing emotions from female faces, or that women would be better in recognizing only stereotypical female emotions [20, 21, 22]. These have all received minimal support. One alternative explanation that has been advanced for the inconsistencies in previous studies is the nature of the stimuli: women would be particularly better in recognizing subtle emotions, such as when the emotion is less intense or prototypical [21]. This implies that women would be more sensitive to subtle cues of emotional expressions. We refer to this explanation as the *emotional sensitivity hypothesis* [23, 24]. We argue that this sensitivity can be better tapped with an emotional intensity profile task rather than a categorization task.

The present paper reports a test of the emotional sensitivity hypothesis in a large community sample, including six emotions, displayed at different levels of prototypicality and intensity. In addition, we also explore the relation between self-reported emotional intelligence (EI) and actual emotion perception performance. Although some studies have combined EI and emotion perception tasks [25], research to date has to our knowledge rarely combined self-perception and actual emotion perception when examining gender differences.

### Gender differences in facial emotion recognition

There is an abundance of research on sex differences in emotion recognition. Several meta-analyses on gender differences in nonverbal decoding have shown that women are superior in decoding emotions than are men [16, 17, 18]. Of the studies included in these meta-analyses, 80% show a female advantage, although differences were small to moderate. Different explanations have been proposed for this female advantage in nonverbal recognition. Many of these explanations are distal [26, 27], referring to the different social roles and accompanying status positions of men and women, or the biological competence of women to read others’ emotions. Since these early meta-analyses, several new studies have been published [19], testing more proximate explanations, related to different modes of emotional processing in the brain (see e.g [28, 29, 30]), different error ratings [31], attention to the eyes [32], the different facial features of male and female faces, and related emotion attributions [33, 34], and the nature, presentation length and intensity of the stimulus materials [35, 23, 24].

In the current research, we test what we have referred to as the ‘emotional sensitivity hypothesis’, focusing on gender differences in the perception of a profile of emotion intensities. Previous research on gender differences in emotion recognition has mostly used a categorization task, in which participants have to choose the correct emotion on the face. These measures thus involve an all or none rating and have shown small to moderate effects, supporting the general idea that the ability to categorize emotions on others’ faces is a prerequisite for smooth social interactions [36] for both men and women. However, the fact that women more often have social-emotional roles or tasks, both with regard to child care, and romantic relations, as well as in organizations, could imply that they are more focused on and motivated to detect subtle cues of emotions. Therefore, the difference between men and women could be more pronounced when studying the perception of subtler emotional signals.

The emotional sensitivity hypothesis states that women are more sensitive to subtle cues, which implies that they perceive the intended emotion as more intense, but only when the cues are subtle or low intense. This explanation has received support in previous studies examining gender differences in emotion recognition, and suggests that men and women may not differ in recognizing clear, prototypical emotions, but that women are more sensitive to emotional refinements and thus only have an advantage in perceiving less intense, or less prototypical emotion expressions [23, 24]. For example, authors in [23] tested gender differences in emotional faces with different intensities. In two experiments, they found that there were no gender differences in the recognition of emotional faces with most extreme intensity, whereas gender differences were found for lower level intensities. In addition, others [24] provided participants with short videos of morphed faces, starting with neutral (0% emotion) and ending with 100% emotion (including 6 different emotions). Participants had to label the emotion they perceived (accuracy task) and next, they had to indicate when they started seeing the emotion (sensitivity task). The results showed that women were better both in the accurate labeling, particularly of sadness and surprise and they were quicker in detecting anger and disgust.

Whereas both these studies included different intensities of emotion, and thus allowed a test of emotional sensitivity, they also used categorical recognition with a forced-choice paradigm. This response format may be less suitable to detect whether men and women differ in their perception of subtle emotions. Indeed, results in [31] showed participants stimulus faces (from JACFEE: 56 expressions of 7 emotions) with different presentation length and then compared intensity ratings of the correct (target) emotion and the incorrect (non-target) emotion, rather than using a categorical response option. Their results showed that women judged the target emotion as being more present on the face, and the non-target emotion as less present than did men. This was the case for disgust, happiness, sadness and surprise. Interestingly, no gender differences in the ratings of non-target emotions were found. In a second study, shorter presentation times were used (70ms, 130ms, 200ms), which showed that women were overall better than men, only when the presence of different emotions could be rated (scalar ratings), and not when they had to select just one emotion. No interaction between the speed of presentation and gender was found, thus women performed better than men in all three presentation times. Again, women more often rated the target emotions as present, and the non-target emotions as absent, compared to men.

Together, the results of these studies are puzzling and partly contradict each other. Whereas [23] found that women are better in identifying less intense emotions, this was not replicated in [31], where different presentation times and intensity ratings were used. This discrepancy could be due to a ceiling effect, but also to the fact that more fine-grained response options were used in the latter study. The fact that women more often perceived the target emotion to be present than men may further suggest that women are better in distinguishing the intended emotion, among other emotional cues. This difference would become less visible in a forced-choice categorization task than in an emotion intensity profile task where participants have to rate the intensity of several different emotions, which allows the detection of more subtle differences between men and women. In response to the authors’ call for a replication of their findings [31], we test the emotional sensitivity hypothesis by including stimuli of different prototypicality (icons, avatars and human faces), and displaying six emotions with two different intensity levels. We used multiple intensity rating scales, tapping into the perceived intensity profile of different emotions (emotion intensity profile task), which also allows the examination of gender differences in the perception of emotion intensity profiles.

### Relation between self-perception and ability

As noted above, the stereotype that women are the more emotional sex, but also the belief that women are better in dealing with their own and others’ emotions is a prevalent stereotype in the Western world [12]. This stereotype also influences self-perceptions, and indeed, most research on self-reported measures of emotional intelligence (EI), interpersonal sensitivity or empathy shows that women perceive themselves to be more emotional intelligent, interpersonally sensitive and empathic [37]. The question is, whether these perceptions reflect their actual performance, or whether they are merely based on self-stereotypes, which would suggest that we should not trust such self-reports.

There are two research lines in EI research, one advocating an ability model [1], and the other a mixed model, which consider EI as a combination of personality, affect, and a reflection on one’s skills to deal with emotions. This latter model is often considered as an umbrella construct, and is generally measured with self-report questionnaires [38]. However, it has been seriously criticized as flawed and lacking strong empirical support [39]. Despite the fact that self-reports may not reflect the truth about one’s emotional abilities, we believe that such self-perceptions are important to study, either when they show a discrepancy or coherence with actual performance. A previous meta-analysis on the relationship between intelligence and interpersonal sensitivity [40] for example, has shown a small-to-medium effect between comparable constructs. To date, there is no research to our knowledge that has examined gender difference in self-reported EI, as well as emotion perception performance in one study. The question is what the sources of such self-perceptions are, and how and when they are influenced by actual abilities. In the present study, we expect that there will be a correlation between EI and the performance on an emotion perception test, because the ability to perceive and understand others’ emotions can form the input, as well as the consequence of one’s self-perception of emotional intelligence.

### Current research

The current study tests the emotional sensitivity hypothesis of gender differences in emotion perception in a large community sample. We hypothesize that women are better than men at perceiving subtle, i.e. less intense and less prototypical emotions, independently of gender of the target or the type of emotion (Hypothesis 1a). We further hypothesize that women perceive target emotions (i.e. the intended emotions) as more intense than men, whereas we do not expect gender difference in perceiving the intensity of non-target emotions (Hypothesis 1b). In addition, we hypothesize that women believe they are better at dealing with and recognizing emotions, as reflected in a higher score on a self-reported EI test [3] (Hypothesis 2). Finally, we hypothesize that self-reported EI, in interaction with gender should predict the perception of target emotions, but not the perception of non-target emotions (Hypothesis 3).

## Materials and methods

### Participants and design

Six thousand hundred and two participants filled out an on-line questionnaire. We first removed all participants (N = 12) who indicated a 1 or 2 on a 7-point scale measuring self-reported seriousness in participating in the experiment. In addition, we excluded all participants who had not finished the tasks (N = 218), resulting in a total of 5872 (31.9% male; *M*_age_ = 44.76, *SD* = 14.84, 97.5% had Dutch as their primary language). The study was granted permission by the Ethical Committee of the Faculty of Social and Behavioral Sciences.

The study had a 2 (Gender respondent: male, female) by 2 (Intensity: low, high) by 3 (Abstraction: human face, computer generated face, drawn iconic face) by 2 Cognitive Load (yes, no) between-subjects design. Thus, participants saw six emotions (happiness, sadness, anger, surprise, fear, and disgust) at only one intensity level and of one type of abstraction. Furthermore, each participant saw four pictures of each emotion, i.e. 24 pictures in total (see Stimuli for a more elaborate description). We do not report the effects of Cognitive Load here, as this was included for exploratory reasons. This factor did not affect any of the dependent variables, nor did it interact with any of the other factors. The emotion intensity profile task task consisted of rating the intensity of different emotions per face.

### Procedure

The study was part of a cooperation between Dutch television (NTR and VPRO) and two universities (University of Amsterdam and Delft University). Participants were recruited through science programs on television and on the website of the respective broadcasting companies. On the site the research was referred to as a study on social skills and a short description of the overall aim of the research was provided. Interested participants were directed to the questionnaire. Participants participated out of free choice. The study used a web-based tool, NetQ, to present the materials. Participants were first asked to fill in an informed consent form and some demographics. Then, they were presented with various facial expressions in counterbalanced ordering (counterbalance between subjects). Each face was presented as long as the subject wanted and the subject could then rate the extent to which they thought each of the 6 emotions was present in the face. A 6-point rating scale ranging from 0 (*not present*) to 5 (*strongly present*) was used, reflecting a judgement of perceived intensity of each of the emotions. This resulted in six ratings for each of the 24 faces. The study took approximately 20 minutes. Subjects could also click the option ‘no emotion present’.

### Materials

We included 6 emotions: happiness, anger, sadness, fear, surprise, and disgust. Each emotion was shown by 4 different models (2 male, 2 female), and thus each participant rated the intensity of six emotion labels for emotions expressed by 24 models (see S1 Instructions and Questionnaires). Three different types of stimuli were used as a between-subjects factor, varying in abstraction. The human faces were stills taken from short clips from a previously validated database of human facial expressions, the ADFES [41]. The computer generated faces (avatars) were stills taken from animations from a set of previously validated expressions based on FACS [42], developed by one of the researchers (JB). The iconic facial expressions were drawn by one of the researchers in the project, based on general FACS guidelines. Generation of these three sets of stimuli was independent, i.e., none of the researchers was involved in the generation of one of the other sets. The different intensity levels were constructed by manipulating the intensity of the most prototypical action unit for each emotion (AU12, smile for happiness; AU4, frown for anger; AU1and4, and AU14, mouth corner lowering for sadness; AU9, nose wrinkles for disgust; AU1, 2 and 5, eye widening and raised eyebrows for surprise). These action units were either depicted as more intense, as in the case of the icons, or manipulated stronger, as in the case of the avatars. In the case of the human pictures, we took stills from short film clips that started with neutral and ended with a full-blown emotion (apex). The low intensity stills were taken from an earlier frame in the video clip than the high intensity stills (see S1 Exemplar stimuli). The avatars and icons used different intensities of the prototypical action units (e.g., a stronger frown in the anger display).

In addition to the emotion perception task, we included a self-report Emotional Intelligence questionnaire developed by [10] in order to explore its relation with actual performance. The 33-item measure has a good internal test-retest reliability and has shown to correlate with other constructs related to EI, including alexithymia, attention to feelings, clarity of feelings, mood repair, optimism, impulse control and mental health [25, 43].

We added 5 items to this questionnaire that would directly tap into the self-reported ability to recognize specific emotions, namely ‘I do not always know how others feel’ (reverse coded); ‘I immediately notice when someone is irritated’; ‘I always pay more attention to what people say than how they look’ (reverse coded); ‘I often see when people have experienced something sad’; ‘I can tell from someone’s face if he or she is nervous’. We also included the Sense of Power scale [44], but due to space constraints, we do not report the effects here. Readers who are interested in this, can contact the author. Finally, we asked participants how seriously they had been in filling in the questionnaire. In order to control for possible priming effects, the sequence of questionnaires and face ratings was counterbalanced, with half of the respondents first filling in the questionnaires and the other half first rating the faces.

## Results

We analyzed the data with SPSS, version 22. We first examined whether male and female participants were equally serious in their engagement in the task. Female participants were slightly more serious (*M* = 6.42, *SE* = .012) than male participants (*M* = 6.37; *SE* = .017), *F* (1, 5870) = 5.307, *p* = .021, *η*^2^ = .001. If we only select the condition for the human faces (and exclude the avatars and icons conditions), the difference becomes non-significant, *F* (1, 2053) = 2.113, *p* = .146.

### Intensity ratings of target and non-target emotions at different levels of abstraction and intensity

We first computed ‘target’ and ‘non-target’ emotion indices for each emotion (e.g., the perceived intensity of sadness for a sad face was calculated as the *target* emotion intensity, whereas the average perceived intensity of all non-target emotions—happiness, fear, anger, surprise and disgust in the case of sadness—was computed as the *non-target* emotion intensity). So, the target emotion rating is operationalized as the perceived intensity of the intended emotion display, and the non-target emotion rating is the average perceived intensity of the non-intended emotion displays. This was calculated across male and female models.

We first conducted an ANOVA with Gender, Abstraction, and Intensity as factors on *target* emotion ratings. We found no main effect of Gender, *F* (1, 5860) = 1.96, *p* = .161, nor any interaction effects with Gender (all *F*’s < 2.83). We found a significant effect of Intensity, *F* (1, 5860) = 636.960, *p* < .0001, *η*^2^ = .049, and of Abstraction, *F* (1, 5860) = 302.825, *p* < .0001, *η*^2^ = .046. (We also found a significant interaction between Intensity and Abstraction, *F* (1, 5860) = 58.353, < .0001, *η*^2^ = .009, which is of no further interest for this paper). The means (see Table 1) show that high intensity faces are rated as more intense, and that human faces are rated as more intense than icons, which are rated as more intense than avatars. We also conducted an ANOVA with Gender, Abstraction, and Intensity as factors on *non-target* emotion ratings. We found a significant main effect of Gender, *F* (1, 5860) = 75.512, *p* < .0001, *η*^2^ = .011, an interaction with Abstraction, *F* (1, 5860) = 3.952, *p* = .019, *η*^2^ = .001, but not the expected interaction with intensity, *F* (1, 5860) = .000, *p* = .996. We further found a main effect of Intensity, *F* (1, 5860) = 53.587, *p* < .0001, *η*^2^ = .008, and a main effect of Abstraction, *F* (1, 5860) = 182.984, *p* < .0001, *η*^2^ = .057. We did not find an interaction between Intensity and Abstraction, *F* (1, 5860) = .479, *p* = .62. Post-hoc tests show that men rate the non-target emotions as more intense than women in human faces, *F* (1, 2054) = 76.271, *p* < .0001, *η*^2^ = .031, in avatars *F* (1, 1893) = 17.785, *p* < .0001, *η*^2^ = .009, and in icons, *F* (1, 1919) = 9.919, *p* = .002, *η*^2^ = .005 (see Table 1 for the means).

**Table 1:** Mean (and standard error) of the perceived intensity of target and non-target emotions.

	Target		Non-Target	
Abstraction	Men	Women	Men	Women
	N = 1873	N = 3999	N = 1873	N = 3999
**Human** (N = 2055)				
High intense (N = 1071)	3.90 (.039)	3.99 (.026)	.47 (.019)	.33 (.013)
Low intense (N = 984)	3.16 (.039)	3.16 (.028)	.54 (.020)	.41 (.014)
**Avatar** (N = 1894)				
High intense (N = 968)	3.22 (.040)	3.22 (.028)	.46 (.028)	.38 (.020)
Low intense (N = 926)	2.80 (.041)	2.73 (.028)	.56 (.029)	.50 (.020)
**Icon** (N = 1923)				
High intense (N = 981)	3.56 (.041)	3.63 (.027)	.50 (.029)	.42 (.019)
Low intense (N = 942)	3.29 (.041)	3.36 (.028)	.50 (.029)	.39 (.020)
Total (N = 5872)	3.32 (.016)	3.35 (.011)	.47 (.012)	.36 (.008)

### Different intensity ratings per emotion for human faces

Because the largest gender differences were found for human stimuli, and because previous research is largely based on human faces, we further focus on human faces for the subsequent analyses (*N* = 2055).

An ANOVA with Gender and Intensity on target ratings, showed no effect of Gender, *F* (1, 2051) = 1.967, *p* = 1.61, and no significant interaction, *F* (1, 2051) = 1.641, *p* = .200, but an effect of Intensity, *F* (1, 2051) = 545.252, *p* < .0001, *η*^2^ = .209. A similar ANOVA on the non-target ratings showed a main effect of Gender, *F* (1, 2051) = 66.597, *p* < .0001, *η*^2^ = .031, an effect of Intensity, *F* (1, 2051) = 20.619, *p* < .0001, *η*^2^ = .010, and again no significant interaction, *F* (1, 2051) = .424, *p* = .515. In other words, the absence of interactions between intensity and gender implies that gender differences in perceiving target emotions were not larger for less intense emotions.

#### Target emotions

In order to examine whether the intensity rating of target and non-target emotions is different for the type of emotion, we first examined whether male and female models were differently perceived, by performing a repeated measure ANOVA on the target emotions displayed by male versus female models. There was a significant difference, *F* (1, 2053) = 106.681, *p* < .0001, *η*_p_^2^ = .049, with female models (*M* = 3.644, *SE* = .027, whose expressions were rated as more intense than those of male models *M* = 3.505, *SE* = .028, however, this was not qualified by a difference between male and female participants, *F* (1, 2053) = .000, *p* = .995.

We then conducted a MANOVA with Gender and Intensity on the six target emotions. We found a main effect of Gender, *F* (6, 2042) = 2.71, *p* = .013, *η*_p_^2^ = .008. The univariate analyses (see Table 2) show that the Gender effect is only significant for disgust and fear, with women rating both emotions as more intense than men (see Table 3 for the Means and Standard Errors). We further found an effect for Intensity, *F* (6, 2042) = 319.849, *p* < .0001, *η*_p_^2^ = .484, showing that all high intense emotion displays were more often perceived as target emotions than all low intense emotion displays (anger: *F* (1, 2051) = 165.444, *p* < .0001, *η*_p_^2^ = .075, fear: *F* (1, 2051) = 196.742, *p* < .0001, *η*_p_^2^ = .088; disgust: *F* (1, 2051) = 20.368, *p* < .0001, *η*_p_^2^ = .010; happiness: *F* (1, 2051) = 1388.481, *p* < .0001, *η*_p_^2^ = .404; sadness: *F* (1, 2051) = 46.186, *p* < .0001, *η*_p_^2^ = .022; surprise: *F* (1, 2051) = 581.378, *p* < .0001, *η*_p_^2^ = .221), but no interaction between Gender and Intensity was found, *F* (6, 2042) = 1.046, *p* = .393.

**Table 2:** Univariate statistics for target and non-target emotions.

Factor	Emotion	Univariate *F*	*p*	*η*_p_^2^
**Target Emotions**				
**Gender**	anger	2.452	.118	.001
	disgust	5.469	.019	.003
	Fear	3.844	.050	.002
	happiness	.312	.577	.000
	sadness	.550	.458	.000
	surprise	.096	.757	.000
**Intensity**	anger	165.446	.000	.075
	disgust	20.368	.000	.010
	fear	196.742	.000	.088
	happiness	1388.481	.000	.404
	sadness	46.186	.000	.022
	surprise	581.378	.000	.221
**Non-Target Emotions**				
**Gender**	anger	59.670	.000	.028
	disgust	78.359	.000	.037
	fear	35.502	.000	.017
	happiness	29.548	.000	.014
	sadness	40.142	.000	.019
	surprise	35.759	.000	.017
**Intensity**	anger	36.087	.000	.017
	disgust	23.599	.000	.011
	fear	5.197	.023	.003
	happiness	91.602	.000	.043
	sadness	3.759	.053	.002
	surprise	32.411	.000	.016

**Table 3:** Means. SE and CI of intensity ratings of target emotions and non-target emotions split by emotion display and gender.

Emotion Display	Gender	Mean	SE	95% Confidence Interval		
				Lower Bound		Upper Bound
**Target Emotions**						
Anger	male	3.912	.038	3.839	3.986	
	female	3.990	.026	3.939	4.041	
Disgust	male	3.461	.044	3.376	3.547	
	female	3.589	.030	3.530	3.647	
Fear	male	2.477	.052	2.375	2.580	
	female	2.610	.036	2.539	2.681	
Happiness	male	3.727	.045	3.639	3.816	
	female	3.771	.031	3.710	3.832	
Sadness	male	3.892	.039	3.815	3.968	
	female	3.864	.027	3.811	3.917	
Surprise	male	3.736	.047	3.643	3.828	
	female	3.736	.033	3.672	3.800	
**Non-Target Emotions**						
Anger	male	.410	.017	.376	.444	
	female	.248	.012	.224	.271	
Disgust	male	.585	.018	.549	.621	
	female	.388	.013	.364	.413	
Fear	male	1.018	.021	.977	1.059	
	female	.866	.014	.837	.894	
Happiness	male	.146	.009	.128	.164	
	female	.086	.006	.074	.098	
Sadness	male	.481	.018	.445	.517	
	female	.339	.013	.314	.364	
Surprise	male	.396	.015	.366	.426	
	female	.285	.010	.265	.306	

#### Non-target emotions

We conducted a similar analysis for all non-target emotions. We again found a significant main effect for Gender, *F* (6, 2046) = 165.446, *p* < .0001, *η*_p_^2^ = .039, and an effect for Intensity, *F* (6, 2042) = 42.598, *p* < .0001, *η*_p_^2^ = .111, but no interaction between Gender and Intensity, *F* (6, 2042) = 1.006, *p* = .420. The univariate analyses (Table 2) show that both the Gender and Intensity main effects were significant for all emotions (see Table 3 for the means and SE): Low intense emotion displays were more often perceived as non-target emotions, and men overall perceived non-target emotions as more intense than did women. Table 4 further reports the ‘confusions’, which in this case implies perceiving traces of other emotions on a face. For example, people also see some anger in disgust faces and vice versa, and they see some fear in surprise faces and vice versa. In addition, the intensity of emotions that are perceived on a face is clearly valence based: we generally do not perceive a lot of happiness in faces displaying negative emotions, nor the other way around.

**Table 4:** Confusion matrix: Means and standard errors for all emotion displays (lines) and intensity ratings (columns).

	Intensity ratings											
	Anger		Disgust		Fear		Happy		Sad		Surprise	
Displays	M	F	M	F	M	F	M	F	M	F	M	F
Anger			.605 .032	.380 .022	.371 .023	.219 .016	.071 .010	.053 .007	.562 .028	.359 .019	.449 .027	.246 .019
Disgust	1.530 .047	1.115 .032			.370 .023	.232 .016	.044 .007	.031 .005	.570 .027	.361 .019	.418 .024	.218 .017
Fear	1.949 .031	1.914 .021	1.002 .043	0.795 .029			.076 .009	.036 .006	2.099 .056	2.173 .037	2.393 .057	2.216 .038
Happy	.067 .009	.041 .006	.080 .009	.036 .006	.144 .013	.098 .009			.201 .015	.148 .010	.245 .017	.119 .012
Sad	.704 .034	.559 .023	.424 .023	.207 .016	.799 .036	.651 .025	.041 .007	.021 .005			.432 .026	.254 .018
Surprise	.181 .015	.118 .010	.212 .015	.111 .011	.829 .035	.688 .024	.482 .026	.356 .018	.285 .018	.165 .013		

In order to further analyze whether specific non-target emotions were rated as more intense by men than women, we conducted six separate MANOVAs with Gender and Intensity as factors and all non-target emotions per emotion display as dependent measure. Here we will report only the main effects of Gender and its interaction with Intensity (univariate effects are reported in Table 5 and other statistics in Table 6). For the *anger* displays, we found a significant main effect of Gender, *F* (5, 2047) = 12.55, *p* < .0001, *η*_p_^2^ = .008, and of Intensity, *F* (5, 2042) = 18.79, *p* < .0001, *η*_p_^2^ = .484, but no interaction between Gender and Intensity, *F* (5, 2042) = 0.498, *p* = .778. Men rated all negative non-target emotions as more intense than women. For the disgust displays, we found a significant main effect of Gender, *F* (5, 2047) = 17.96, *p* < .0001, *η*_p_^2^ = .042, and of Intensity, *F* (5, 2042) = 25.61, *p* < .0001, *η*_p_^2^ = .059, and an interaction between Gender and Intensity, *F* (5, 2042) = 2.71, *p* = .019, *η*_p_^2^ = .007. Men rated all negative non-target emotions as more intense than women. For the *fear* displays, we found a significant main effect of Gender, *F* (5, 2047) = 9.15, *p* < .0001, *η*_p_^2^ = .143, and of Intensity, *F* (5, 2042) = 70.02, *p* < .0001, *η*_p_^2^ = .146, but again no interaction between Gender and Intensity, *F* (5, 2042) = 1.64, *p* = .147. Men rated all non-target emotions as more intense than women. For the *happy* displays, we found a significant main effect of Gender, *F* (5, 2047) = 8.95, *p* < .0001, *η*_p_^2^ = .021, and of Intensity, *F* (5, 2042) = 27.92, *p* < .0001, *η*_p_^2^ = .064, and no interaction between Gender and Intensity, *F* (5, 2042) = 1.76, *p* = .118. Men rated all non-target emotions as more intense than women. For the sad displays, we found a significant main effect of Gender, *F* (5, 2047) = 13.39, *p* < .0001, *η*_p_^2^ = .032, and of Intensity, *F* (5, 2042) = 6.54, *p* < .0001, *η*_p_^2^ = .016, but no interaction between Gender and Intensity, *F* (5, 2042) = 1.88, *p* = .094. Men rated all non-target emotions as more intense than women. Finally, for the surprise displays, we found a significant main effect of Gender, *F* (5, 2047) = 8.96, *p* < .0001, *η*_p_^2^ = .021, and of Intensity, *F* (5, 2042) = 28.15, *p* < .0001, *η*_p_^2^ = .064, and again no interaction between Gender and Intensity, *F* (5, 2042) = 2.11, *p* = .062. Men rated all non-target emotions as more intense than women.

**Table 5:** Univariate effects for all non-target emotions per emotion display.

Displays	Factors	Non-target Emotion	*F*	*p*	*η*_p_^2^
Anger	Gender	fear	30.993	.000	.015
		happiness	2.206	.138	.001
		surprise	38.402	.000	.018
		sadness	36.945	.000	.018
		disgust	33.126	.000	.016
	Intensity	fear	14.094	.000	.007
		happiness	1.529	.216	.001
		surprise	54.621	.000	.026
		sadness	44.384	.000	.021
		disgust	3.189	.074	.002
Fear	Gender	anger	30.317	.000	.015
		happiness	13.428	.000	.007
		surprise	17.296	.000	.008
		sad	17.337	.000	.008
		disgust	9.712	.002	.005
	Intensity	anger	77.487	.000	.036
		happiness	19.867	.000	.010
		surprise	60.514	.000	.029
		sad	.050	.822	.000
		disgust	93.203	.000	.043
Sadness	Gender	anger	12.418	.000	.006
		fear	11.140	.001	.005
		surprise	32.216	.000	.015
		happiness	5.793	.016	.003
		disgust	62.206	.000	.029
	Intensity_	anger	.420	.517	.000
		fear	18.018	.000	.009
		surprise	.485	.486	.000
		happiness	4.861	.028	.002
		disgust	3.616	.057	.002
Happiness	Gender	anger	5.951	.015	.003
		fear	9.413	.002	.005
		surprise	37.159	.000	.018
		sad	8.445	.004	.004
		disgust	17.647	.000	.009
	Intensity	anger	39.374	.000	.019
		fear	42.532	.000	.020
		surprise	12.475	.000	.006
		sad	126.837	.000	.058
		disgust	53.364	.000	.025
Disgust	Gender	fear	24.422	.000	.012
		happiness	2.222	.136	.001
		surprise	46.228	.000	.022
		sad	39.346	.000	.019
		Anger	53.793	.000	.026
	Intensity	fear	.615	.433	.000
		happiness	.230	.632	.000
		surprise	9.812	.002	.005
		sad	25.532	.000	.012
		anger	73.651	.000	.035
	Gender * Intensity	fear	2.099	.148	.001
		happiness	2.151	.143	.001
		surprise	7.389	.007	.004
		sad	.547	.460	.000
		anger	.631	.427	.000
Surprise	Gender	anger	12.305	.000	.006
		fear	10.840	.001	.005
		sad	29.674	.000	.014
		happiness	16.093	.000	.008
		disgust	29.261	.000	.014
	Intensity	anger	22.668	.000	.011
		fear	67.661	.000	.032
		sad	55.250	.000	.026
		happiness	11.415	.001	.006
		disgust	2.407	.121	.001

**Table 6:** Means, standard errors, and CI for all non-target emotions per emotion display, split by gender.

**Anger Displays**				95% Confidence Interval	
	Gender	Mean	SE	Lower Bound	Upper Bound
Fear	male	.371	.023	.327	.416
	female	.219	.016	.189	.250
Happiness	male	.071	.010	.052	.090
	female	.053	.007	.040	.067
Surprise	male	.449	.027	.396	.502
	female	.246	.019	.209	.282
Sadness	male	.562	.028	.508	.616
	female	.359	.019	.322	.396
Disgust	male	.605	.032	.542	.668
	female	.380	.022	.337	.423
**Disgust Displays**					
Anger	male	1.530	.047	1.439	1.621
	female	1.115	.032	1.052	1.178
Fear	male	.370	.023	.325	.415
	female	.232	.016	.201	.263
Happiness	male	.044	.007	.030	.059
	female	.031	.005	.021	.041
Sadness	male	.570	.027	.516	.624
	female	.361	.019	.324	.398
Surprise	male	.418	.024	.371	.466
	female	.218	.017	.186	.251
**Fear Displays**					
Anger	male	1.006	.035	.938	1.074
	female	.775	.024	.729	.822
Happiness	male	.085	.008	.068	.101
	female	.047	.006	.036	.059
Surprise	male	2.893	.051	2.792	2.994
	female	2.633	.035	2.564	2.702
Sadness	male	.621	.029	.563	.678
	female	.471	.020	.432	.511
Disgust	male	.928	.039	.853	1.004
	female	.782	.027	.730	.834
**Sadness Displays**					
Anger	male	.704	.034	.638	.771
	female	.559	.023	.513	.605
Fear	male	.799	.036	.727	.870
	female	.651	.025	.602	.700
Surprise	male	.432	.026	.381	.483
	female	.254	.018	.219	.288
Happiness	male	.041	.007	.028	.055
	female	.021	.005	.012	.030
Disgust	male	.424	.023	.379	.468
	female	.207	.016	.177	.238
**Happiness Displays**					
Anger	Male	.067	.009	.050	.084
	Female	.041	.006	.030	.053
Disgust	Male	.080	.009	.063	.097
	Female	.036	.006	.025	.48
Fear	Male	.144	.013	.120	.169
	Female	.098	.009	.081	.115
Sadness	Male	.201	.015	.171	.230
	Female	.148	.010	.127	.168
Surprise	Male	.245	.017	.211	.278
	Female	.119	.012	.096	.124

#### No emotion ratings

We also conducted an ANOVA on the frequency with which male and female participants had marked the ‘no emotion’ option. We found a main effect of Gender, *F* (1, 2051) = 11.071, *p* = .001, *η*^2^ = .006, and of Intensity, *F* (1, 2051) = 27.52, *p* < .0001, *η*^2^ = .013, and no interaction. *F* (1, 2051) = 2.148, *p* = .143. The means show that men (*M* = .878, *SE* = .046) more often think there is no emotion to be perceived than do women (*M* = .686, *SE* = .032). In addition, low intensity displays more often are perceived as showing no emotion (*M* = .930, *SE* = .040) than high intensity displays (*M* = .635, *SE* = .039).

#### Bayesian statistics

In order to test whether the null hypothesis, indicating an absence of gender differences in perception of target, non-target and no-emotions would be more likely than the alternative hypothesis, we conducted Bayesian t-tests. In contrast with frequentist approaches where a hypothesis is rejected on the basis of the *p*-value, Bayesian testing provides the ratio of likelihoods, given the null hypothesis versus the alternative hypothesis. In other words, this approach gives answer to the question how likely it is that there are no gender differences versus there are. The so-called Bayes factors represent this likelihood. Here, the Bayes factors were respectively BF_01_ = 4.307 (target emotions), BF_01_ = 369.726 (non-target emotions), and BF_01_ = 195.5 (no-emotions), all indicating substantial evidence for the null hypothesis, in this case, the absence of a gender difference [45].

### Emotional intelligence as predictor of emotion perception

For the respondents who only rated the human faces, we first calculated the reliability of the EI scale with (Cronbach’s α = .912) and without the additional 5 items (Cronbach’s α = .866). Because there were no differences between the analyses with the extended EI test and the original one, we used the original test in the subsequent analyses. (An ANOVA with Gender on the extended EI test also showed that women had small, but significantly, higher EI scores (*M* = 5.16, *SE* = .017) than men (*M* = 4.82; *SE* = .025), *F* (1, 2026) = 123.46, *p* < .0001). An ANOVA with Gender showed that women had significantly higher EI scores (*M* = 4.91, *SE* = .016) than men (*M* = 4.64; *SE* = .023), *F* (1, 2049) = 90.26, *p* < .0001, *η*^2^ = .04. Applying Bayesian statistics in order to test the reliability of the null hypothesis, shows a Bayes factor of BF_01_ = 1.161, implying only anecdotal evidence for the null hypothesis.

We then conducted a multiple regression analysis with EI, Gender and the Interaction term as predictors of the total of target ratings across emotions as the dependent variable. Assumptions of multicollinearity and homoscedasticity were met. The results show that the model is significant (*F* (3, 2047) = 12.791, *p* < .0001, adjusted R^2^ = .017). None of the predictors were significant (Gender: unstandardized *β* = -.220; SE = .284; *t* = -.776, *p* = .438; EI: unstandardized *β* = .088; SE = .096; *t* = .918, *p* = .358; Interaction: unstandardized *β* = .047; SE = .057; *t* = .833, *p* = .405). We also conducted a similar regression with the non-target ratings as the dependent measure. Here, the assumption of homoscedasticity was not met, which makes the analysis unreliable. The results show that the model is significant (*F* (3, 2047) = 23.721, *p* < .0001, adjusted R^2^ = .032). Here, EI is a significant predictor (unstandardized *β* = .087; SE = .042; *t* = 2.057, *p* = .040), as well as its interaction with Gender, unstandardized *β* = -.053; SE = .025; *t* = -2.111, *p* = .035, whereas Gender is not (unstandardized *β* = -.123 (SE = .125); *t* = .988, *p* = .323). These findings indicate that none of the factors predicts the perception of target emotions intensity, whereas the perception of non-target emotions is predicted by both EI and its interaction with gender. However, we should note that the residuals in this regression equation are not equally distributed.

## Discussion

The present study tested the emotional sensitivity hypothesis in a large communal sample. This hypothesis poses that women are not generally better in the detection of emotions on the face, but would be especially better in the perception of target emotions in low intensity and less prototypical emotion displays, whereas no or fewer gender differences would be found for highly intense and prototypical emotion displays. In addition, we tested whether participants’ self-perceived emotional intelligence could explain their emotion perception ratings. The stimuli included a variety of human and non-human faces, displaying six different emotions, with two levels of intensity and posed by both male and female models. These features enabled us to reach reliable conclusions regarding gender differences in emotion perception, a long-standing issue of interest and debate in emotion research. More specifically, we did not use a categorization task, but an emotion intensity profile task (see also [46]), focusing on the perceived intensity of several different emotions.

We did not find any empirical support for gender differences in the perceived intensity of the target emotion displays, either on human faces, avatars or icons, nor in interaction with the intensity of the emotion display. Both men and women generally perceived low intense emotions to be less intense than highly intense emotions, and this applied to the stimuli at all abstraction levels (humans, avatars, icons). Thus, the emotional sensitivity hypothesis was not supported. This applied to the perception of target as well as non-target emotions (e.g., perceived intensity of anger on a sad face). In addition, neither self-perceived emotional intelligence, nor its interaction with gender significantly predicted the perception of target emotions. Men did score lower on self-perceived EI, which suggests that they think of themselves as less confident in perceiving, understanding and regulating emotions than did women. However, this did not affect the intensity ratings of target emotions. In other words, men and women’s self-perceived emotional intelligence is not a reliable predictor of rating the intensity of the intended emotion displays on the face (see also [25]).

Unexpectedly, we found significant gender differences in the perception of non-target emotions, as well as in the perception of an absence of emotions in the face, such that men rated non-target emotions as more intense than did women, and even when there was no emotion at all (neutral faces). This applied to all emotions, but it should be noted that the effect sizes for these differences were very small. These findings are in contradiction with [31], who also used rating scales for several target and non-target emotions presented at different exposure times, and found that women tend to give higher ratings on target emotions, whereas no differences were found for non-target emotions. The findings from this study as well as our own study may suggest that in both studies men seem more inaccurate than women, because they either score lower on target emotions, or higher on non-target emotions. There are several interpretations of this gender difference. One is that men are simply less competent in emotion perception, but our findings partly contradict this, because men did perceive the target emotions to be present as much as women did. An alternative explanation could be that men are more focused on subtle facial expressions, and thus perceive more complex emotion profiles on the face. This would suggest that men are better in perceiving emotional complexity. Still another interpretation could be that men are more uncertain about their emotion perception, and get more easily confused when asked to rate the intensity of several emotions (see also [47]). This may have resulted in perceiving trails of the presence of other emotions as well. The latter explanation is would be in line with the result that EI and its interaction with gender significantly predicted the perception of non-target emotions, suggesting that men’s lower scores on EI accounted for their perception of more intense non-target emotions. However, on the basis of the present data we cannot draw strong conclusions on the validity of these different explanations, because we did not explicitly test them against each other. This could be an interesting venue for future research.

The results of this study that there are no gender differences in the perception of target emotions diverge from various earlier reviews and meta-analyses on gender differences in emotion accuracy [16, 17, 18, 19, 48] and therefore demand an explanation. One explanation is that the studies in which no differences were reported were not included in these meta-analyses, leading to an overestimation of gender differences. All though this could be seen as a file drawer effect, this is not necessarily the case, because not all reported research aims to study gender differences, and therefore do not report them. A second explanation may refer to the stimuli used in our study, which may have been not subtle enough to show differences. The low intensity stimuli of human faces, however, were very subtle, as they also resulted in the perception of non-target emotions, and thus we do not believe that these stimuli were too easy to perceive [49]. Further, we included different types of stimuli, and these stimuli therefore seem to be fairly representative and generalizable, in contrast with studies in which only one set of faces has been used.

A third explanation relates to the use of intensity ratings rather than forced choice accuracy scores, which were used in most previous studies. An inspection of the means of target and non-target emotions, however, shows that the target emotion ratings are much higher than the non-target emotions, such that the first set of ratings can easily be interpreted as the recognition of the ‘correct’ emotions. The expected advantage was that such ratings would enable us to detect subtler differences in what men and women perceive in others’ faces (see also [31]). Rather than scoring a hit or miss, we were able to examine whether men and women differed in the perception of a range of emotions on a face. Thus, we do not think that intensity ratings have obscured gender differences. On the contrary, one would expect more rather than fewer differences with this emotion intensity profile task.

A fourth explanation concerns the sample. Of course, our sample is not completely representative, as participants voluntarily participated in this research through advertisements on a website and on television. However, we think this sample is less biased than in many previous studies, which used student samples. The current sample is a communal sample with participants of various age groups and educational background. We would even expect more gender differences in a non-student sample, because of age and differences in background. We tested this explanation (not reported here), but did not find any significant interaction between gender and age or gender and education level. It could also be that this Dutch sample is different from the US samples used in a majority of the studies. There is no reason to expect that there are huge cross-cultural differences in gender differences in emotion perception, between the Netherlands and the US. Obviously, this assumption should be tested in cross-cultural research, using the same task and stimuli. Another, fifth, explanation of our lack of gender differences may relate to our between-subjects design. Participants only saw low or high intense displays (and only humans, avatars, or icons), and thus no comparison could be made between different ratings of stimuli for the same participants. We do not think that this type of design has led to an absence of differences, however, because this disadvantage only applies when there is huge individual variability in the measures. This was not the case in the present study.

Still one limitation of the present study may have been that we used an online questionnaire, and therefore people may not have paid sufficient attention to the task. In judgments of the seriousness in which they had engaged in the study, however, no significant differences were found between men and women, so this rules out a lack of engagement explanation of why we did not find significant differences. It is clear that the study attracted more female than male participants, which is the case with most research participation, but the high number of participants in the present study makes the data more reliable and representative than in a small student sample.

In sum, on the basis of this study, which includes a large community sample, we have reason to doubt that there are robust gender differences in whether and to what extent they perceive specific emotions to be present in the face. Men do have less confidence in their own emotional intelligence, including their own ability to perceive emotions on a face, than do women. However, this lower score does not predict their perception of target emotions, but it is associated with their stronger perception of trails of emotions that were not intended, or even not present. We should keep in mind, however, that the differences that we found were small, and therefore we cannot yet speculate on social implications of these findings. More research is needed on how men and women exactly differ in their perception of subtle emotion cues. We think it is very important to gain more insight in this process, because in most daily life situations emotional cues are not so clear and straightforward as in experimental research.

## Supporting information

S1 Instructions and Questionnaires (DOCX)

S1 Exemplar Stimuli (DOCX)
