# Supplementary material for: Gender differences in emotion perception and self-reported emotional intelligence: A test of the emotion sensitivity hypothesis
Source: PLoS One. 2018 Jan 25;13(1):e0190712. doi: 10.1371/journal.pone.0190712 (PMC5784910; doi:10.1371/journal.pone.0190712)
Supplement: S1 Instructions and Questionnaires — (DOCX) [file pone.0190712.s001.docx]

**Supporting Information File 1 Instructions and questionnaires**

**Dutch instruction and questionnaire**

Hartelijk welkom bij dit publieksonderzoek!

In de volgende test krijgt u steeds een gezicht te zien waarop een expressie te zien is.

We vragen u aan te geven hoe sterk de intensiteit van de emotie op elk gezicht is. U kunt steeds één of meerdere emoties aankruisen die u denkt te zien in het gezicht, en vervolgens aangeven hoe sterk u denkt dat die emotie is. De emoties waaruit u kunt kiezen staan onderaan in beeld (evenals de mogelijkheid dat er helemaal geen emotie te zien is). Denk niet te lang na, de emotie verdwijnt na 4 seconden van het scherm, dus volg uw eerste indruk.

Hierna volgt er ook nog een vraag over de natuurlijkheid van de expressie: in hoeverre vindt u de emotie die u ziet ‘echt’ of natuurlijk?

Wat voelt deze persoon?

Helemaal niet heel sterk

0 1 2 3 4 5

1. blij
2. boos
3. walging
4. bang
5. verdrietig
6. verbaasd
7. geen emotie

Helemaal niet heel natuurlijk

Hoe natuurlijk vindt u deze expressie? 1 2 3 4 5

**Emotionele Intelligentie (Schutte) (Extra items in italics)**

Deze vragenlijst meet persoonlijkheidsverschillen in emotionele effectiviteit. We vragen u zo eerlijk mogelijk aan te geven of een uitspraak op u van toepassing is.

1. Ik weet wanneer ik met anderen over mijn persoonlijke problemen kan praten
2. Als ik problemen tegenkom, onthoud ik de keren dat ik vergelijkbare problemen tegenkwam en ze overwon.
3. Ik verwacht dat ik goed presteer in de meeste dingen waaraan ik begin
4. Andere mensen nemen mij makkelijk in vertrouwen
5. Ik vind het moeilijk om de emoties van andere mensen te herkennen*
6. Een aantal zeer belangrijke gebeurtenissen in mijn leven hebben ertoe geleid dat ik opnieuw nadenk over wat belangrijk en onbelangrijk is
7. *Ik heb lang niet altijd door hoe andere mensen zich voelen REV**
8. Als mijn stemming verandert, zie ik nieuwe mogelijkheden
9. Emoties behoren tot de zaken die mijn leven de moeite waard maken
10. *Ik let altijd meer op wat mensen zeggen dan op hoe ze kijken REV**
11. Ik ben mij bewust van mijn emoties als ik ze ervaar
12. Ik verwacht dat er goede dingen gebeuren
13. *Ik zie het meteen wanneer iemand geirriteerd is*
14. Ik vind het prettig om mijn emoties met anderen te delen
15. Als ik een positieve emotie ervaar, weet ik hoe ik deze vast kan houden
16. Ik organiseer gebeurtenissen waar anderen plezier aan beleven
17. Ik probeer dingen te doen waar ik blij van word
18. Ik ben mij bewust van de emotionele signalen die ik uitzend
19. Ik presenteer mijzelf op zo’n manier dat ik een goede indruk maak op anderen
20. Als ik in een goede bui ben, is het oplossen van problemen makkelijk voor me
21. Als ik naar de gezichten van anderen kijk, herken ik de emoties die zij ervaren
22. Ik weet waarom mijn emoties veranderen
23. Als ik in een positieve bui ben, ben ik in staat om op goede ideeen te komen
24. Ik heb controle over mijn emoties
25. *Ik merk het vaak wel op als mensen iets verdrietigs hebben meegemaakt*
26. Ik herken mijn emoties gemakkelijk als ik ze ervaar
27. Ik motiveer mezelf door me de positieve uitkomst voor te stellen van taken die ik oppak
28. Ik complimenteer anderen als ze iets goed hebben gedaan
29. Ik ben mij bewust van de emotionele signalen die andere mensen versturen
30. Als iemand mij vertelt over een belangrijk gebeurtenis in zijn of haar leven, voelt het haast alsof ik het zelf heb meegemaakt
31. Als ik een verandering in mijn emoties voelt, kom ik ook vaak met nieuwe ideeen
32. Als ik voor een uitdaging sta, geef ik het op omdat ik denk dat het niet zal lukken*
33. Ik weet wat anderen voelen door slechts naar hen te kijken
34. Ik help andere mensen zich beter voelen als zij in een dip zitten
35. Ik gebruik mijn positieve stemming om te blijven proberen als ik problemen tegenkom
36. Ik weet hoe anderen zich voelen door naar hun stem te luisteren
37. Ik vind het moeilijk om te begrijpen waarom anderen zich op een bepaalde manier voelen*
38. *Ik zie aan iemands gezicht dat hij of zij zenuwachtig is.*

Laatste vragen op het einde:

Tot slot willen we u nog een aantal vragen stellen:

1. Wat is uw geslacht? Man vrouw

2. Wat is uw geboortedatum?

3. Wat is uw nationaliteit?

4. Wat is de nationaliteit van uw moeder? Wat is de nationaliteit van uw vader?

5. Wat is uw hoogst genoten opleiding?

6. Wat is uw beroep?

7. Wat is uw huidige functie?

8. Heeft u deze test serieus ingevuld? 1=helemaal niet; 7=zeer zeker

**Questionnaire translated in English**

What does this person feel?

Stimulus 1 (the stimulus disappears after 4 seconds, or earlier when the respondent clicks on one of the labels)

Not at all very much

0 1 2 3 4 5

1. happy
2. angry
3. disgusted
4. afraid
5. sad
6. surprised
7. no emotion

Not at all Very natural

How natural did you find this expression? 1 2 3 4 5

**The same procedure for the next 31 stimuli**

Last screen: questions at the end:

We finally want to ask you some general questions:

1. Gender? Man vrouw

2. Date of Birth?

3. Nationality?

4. Nationality of your mother? Nationality of your father?

5. What is your highest education?

6. What is your profession?

7. What is you current work?

8. Did you seriously fill in this test? 1=not at all; 7=very
