# Supplementary material for: Gender differences in emotion perception and self-reported emotional intelligence: A test of the emotion sensitivity hypothesis
Source: PLoS One. 2018 Jan 25;13(1):e0190712. doi: 10.1371/journal.pone.0190712 (PMC5784910; doi:10.1371/journal.pone.0190712)

**Supporting Information File 1 Exemplar Stimuli**

Humans High Humans Low


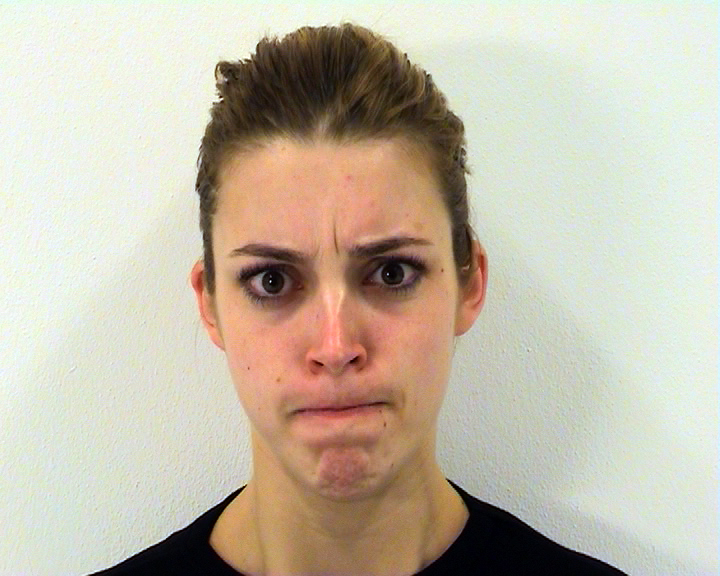

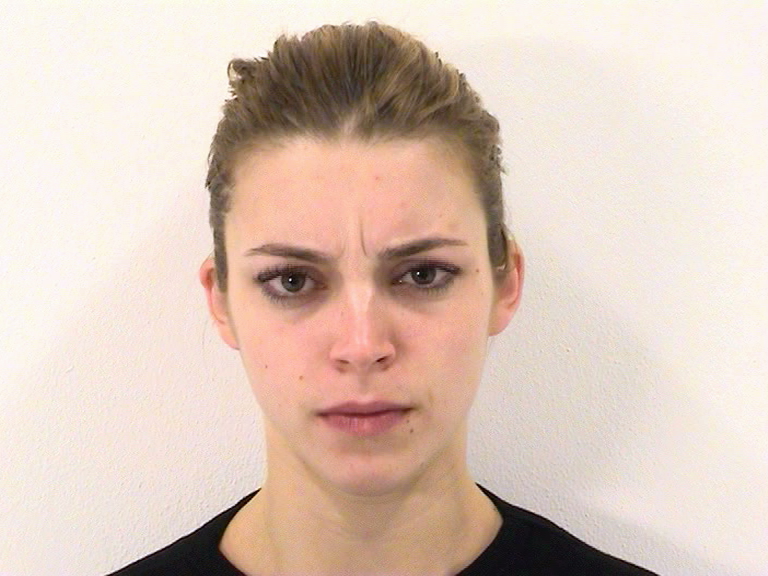


Avatar high Avatar low


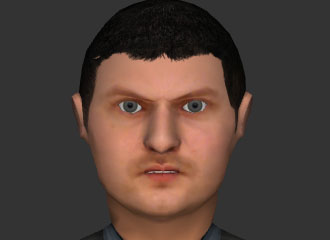

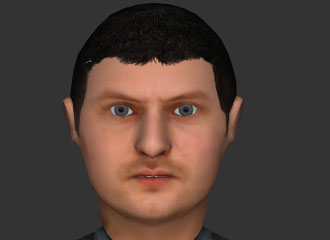


Icons high Icons low


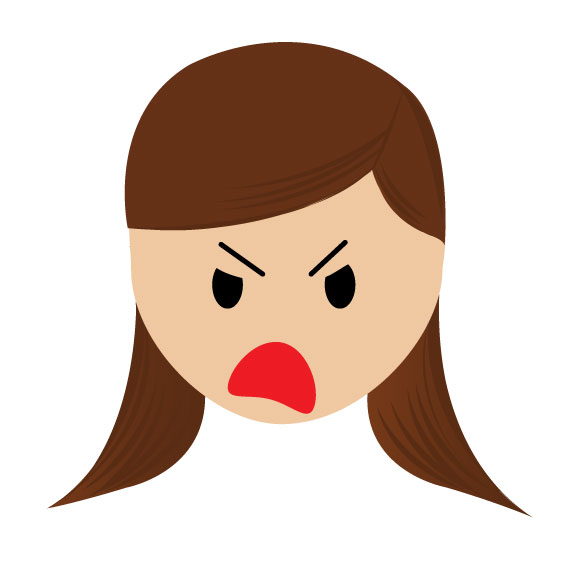

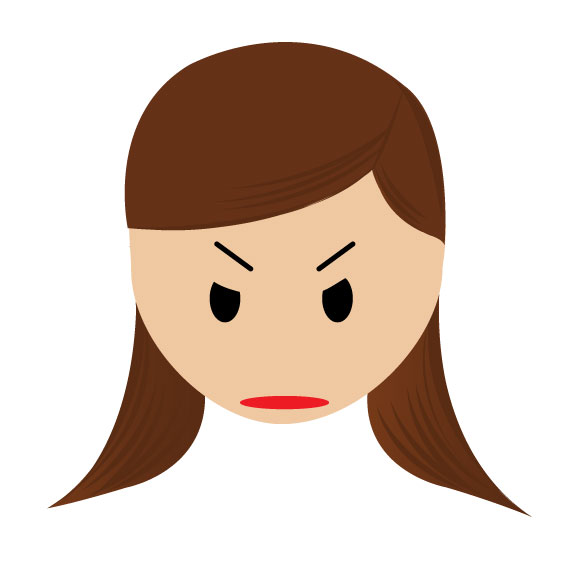

Supplement: S1 Exemplar Stimuli — (DOCX) [file pone.0190712.s002.docx]
